# Understanding COVID-19 Vaccine Hesitancy: A Neuroscientific Protocol

**DOI:** 10.3390/brainsci15060563

**Published:** 2025-05-24

**Authors:** Francesca Pisano, Simona Massimino, Giuseppe Craparo, Gabriella Martino, Francesco Tomaiuolo, Vanni Caruso, Alessio Avenanti, Carmelo Mario Vicario

**Affiliations:** 1 Department of Cognitive Science, University of Messina, 98121 Messina, Italy; frpisano@unime.it (F.P.); cvicario@unime.it (C.M.V.); 2 Faculty of Human and Social Sciences, Kore University of Enna, 94100 Enna, Italy; giuseppe.craparo@unikore.it; 3 Department of Clinical and Experimental Medicine, University of Messina, 98122 Messina, Italy; gabriella.martino@unime.it (G.M.); francesco.tomaiuolo@unime.it (F.T.); 4 School of Psychological Sciences, Psychology, University of Tasmania, Launceston, TAS 7248, Australia; vanni.caruso@utas.edu.au; 5 Centro Studi e Ricerche in Neuroscienze Cognitive, Dipartimento di Psicologia “Renzo Canestrari”, Università di Bologna, Campus di Cesena, 47521 Cesena, Italy; alessio.avenanti@unibo.it; 6 Centro de Investigación en Neuropsicología y Neurociencias Cognitivas, Universidad Católica del Maule, Talca 3460000, Chile

**Keywords:** COVID-19, vaccine hesitancy, psychophysiology, embodiment, motor evoked potentials, electromyography, skin conductance response, virtual reality, psychological traits, research methods

## Abstract

**Background:** Vaccine hesitancy (VH) is a significant public health challenge, especially during the COVID-19 pandemic. Despite extensive research on the psychological and socio-political determinants of VH, its psychophysiological mechanisms remain unexplored. Grounded in the Somatic Marker Hypothesis, this study aims to investigate the neurophysiological and affective processes underlying VH. **Methods:** Two experiments will assess sensorimotor resonance and affective processes in VH. In the first experiment, motor-evoked potentials (MEPs) will be recorded from the deltoid and extensor carpi radialis muscles while participants view images of people receiving COVID-19 and influenza vaccines, as well as blood injections (Block 1), and images of vial containing the same substances (Block 2). Facial electromyographic (EMG) activity will measure disgust and fear responses. In the second experiment, skin conductance response (SCR) will be recorded during a virtual reality-based fear conditioning and extinction paradigm. **Expected Outcomes:** We hypothesize that vaccine-hesitant individuals will exhibit altered sensorimotor resonance, higher affective responses to vaccination stimuli, and impaired fear extinction learning. Psychological traits such as disgust sensitivity, paranoia, anxiety, and dogmatism are expected to be associated with VH. **Conclusions:** By identifying the psychophysiological mechanisms of VH, this study will contribute to developing effective vaccine promotion strategies to address future public health emergencies.

## 1. Introduction

The COVID-19 pandemic has caused a profound transformation in the global healthcare landscape, resulting in over 5.6 million deaths [1]. It has also disrupted social and economic activities worldwide, affecting people’s mental health [2,3]. In response to the rapid spread of the virus, the global community prioritized the development of effective safety measures, with vaccines emerging as a central strategy to control the pandemic [4]. However, despite the availability of COVID-19 vaccines, vaccine hesitancy (VH)—defined as delay or refusal to accept the vaccine—has become a significant global challenge during COVID-19 pandemic [5,6]. The World Health Organization (WHO) identified VH as one of the top public health threats, with estimates suggesting that approximately 26% of the population exhibited resistance to the COVID-19 vaccination [7,8]. A comprehensive approach to addressing VH requires integrating demographic, social, environmental and psychological dimensions [7,9,10,11,12]. Examining these factors is important to combat hesitancy toward the COVID-19 vaccine, guide targeted interventions that sustain effective public health responses, and contribute to managing future pandemics [13]. For example, a systematic review of 23 studies identified key demographic and socioeconomic features influencing COVID-19 vaccine uptake [14]. Older age, higher income, and greater educational level were associated with higher vaccine acceptance [6,15]. Concerns about vaccine safety, distrust in pharmaceutical companies and governmental health policies, absence of prior influenza vaccination, and exposure to misinformation have been strongly linked to VH [6,16,17]. Moreover, religiosity has been associated with lower vaccine intent, particularly in communities were vaccine acceptance conflicts with cultural or religious beliefs [18]. From a psychological perspective, a large body of evidence has reported that individuals with paranoid tendencies, impulsivity, low conscientiousness or emotional instability exhibit greater VH [7,9,19]. Furthermore, dogmatic thinking patterns and reduced empathic capacity are correlated with increased reluctance toward vaccination [19,20,21]. Complementing these findings, a multinational study across 24 countries identified disgust sensitivity and anxiety toward medical procedures (e.g., injections) as critical psychological drivers of anti-vaccine attitude, alongside adherence to individualistic/hierarchical worldviews [9]. Notably, contextual factors such as needle phobia—prevalent in approximately 10% of the population—and maladaptive fear regulation mechanisms may further entrench avoidance behaviors, exacerbating VH [22]. Beyond psychological and contextual variables, political ideology has emerged as a debated determinant of vaccine attitudes. However, empirical evidence remains inconsistent: while some studies report a correlation between a conservative political orientation and increased VH [23], others find no statistically significant relationship [24].

Despite progress in understanding VH, the psychophysiological mechanisms underlying resistance, in the context of COVID-19, remain largely unexplored. Grounded in the Somatic Marker Hypothesis [25], which posits that bodily states and emotional responses shape decision-making, this study’s goals are as follows:

1. Investigate sensorimotor mapping and affective-autonomic responses in vaccine-hesitant and accepting individuals, assessing emotional and physiological reactions to vaccination-related and unrelated stimuli. We will measure motor evoked potentials (MEPs) from the deltoid muscle—a relevant muscle for vaccine inoculation—and the extensor carpi radialis as a control muscle. Additionally, facial electromyography (EMG) will be recorded to assess fear and disgust responses, providing physiological markers of affective processing in response to these stimuli.
2. Examine affective learning processes in VH through a Pavlovian fear conditioning and extinction paradigm, testing whether hesitant individuals exhibit altered fear acquisition, extinction patterns compared to non-hesitant controls. Importantly, no explicit information about vaccination will be provided during the task, allowing us to probe whether differences in basic fear learning mechanisms—independent of vaccine-related content—might underlie or predict vaccine hesitancy. This approach aimed to identify whether disruptions at any stage of the conditioning process are associated with negative attitudes toward vaccination. Skin conductance response (SCR) will serve as the primary psychophysiological marker of conditioned fear responses.
3. Analyze demographic and psychological characteristics associated with VH by comparing experimental and control group on disgust sensitivity, paranoia, anxiety, empathy, intelligence, personality traits, political orientation, and dogmatism, using validated questionnaires.

## 2. Methods and Analysis

### 2.1. Experiment 1

#### 2.1.1. Participants

A total of 40 healthy participants, aged 18 to 50 years, will be recruited for a randomized-controlled study and categorized based on their attitudes toward COVID-19 vaccination:

- Vaccine-Hesitant/Resistant Group (*n* = 20): Individuals reluctant to receive the COVID-19 vaccine, identified using validated psychometric tools, including the Vaccine Hesitancy Scale [26] and the Vaccine Hesitancy Scale for COVID-19 Vaccination [27].
- Vaccine-Accepting Group (*n* = 20): Individuals who are vaccinated or express no hesitation toward vaccination. They will serve as controls.

Recruitment will be conducted through online advertisements and university mailing lists. To ensure comparability, groups will be matched by demographic variables, including age, gender, and educational level.

#### 2.1.2. Sample Size Calculation

The required sample size was determined a priori using GPower 3.1 [28], targeting the primary statistical analysis: a mixed-model ANOVA with 2 between-subjects groups (Vax vs. No-Vax) and 2 within-subjects factors—muscle (deltoid vs. extensor carpi radialis) and condition (Blood, COVID-19, Influenza). The analysis aimed to detect a medium effect size (f = 0.25), considered the smallest scientifically meaningful effect size [29], with a power of 0.95 and α = 0.05. Given the repeated-measures structure, the analysis assumed a moderate correlation (r = 0.5) between the six repeated measurements (2 muscles × 3 conditions), which reflects the expected level of association in related psychometric constructs such as disgust sensitivity and anxiety. This correlation value was specified in GPower to ensure accurate estimation. Under these parameters and assuming ideal sphericity (ε = 1), the required sample size was estimated at 40 participants.

### 2.2. Experiment 2

#### 2.2.1. Participants

A total of 40 healthy participants, aged 18 to 50 years, will be recruited for a randomized-controlled study and categorized based on their attitudes toward COVID-19 vaccination:

- Vaccine-Hesitant/Resistant Group (*n* = 20): Individuals reluctant to receive the COVID-19 vaccine, identified using validated psychometric tools, including the Vaccine Hesitancy Scale [26] and the Vaccine Hesitancy Scale for COVID-19 Vaccination [27].
- Vaccine-Accepting Group (*n* = 20): Individuals who are vaccinated or express no hesitation toward vaccination. They will serve as controls.

Recruitment will be conducted through online advertisements and university mailing lists. To ensure comparability, groups will be matched by demographic variables, including age, gender, and educational level.

#### 2.2.2. Sample Size Calculation

The sample size was determined a priori through power analysis using GPower 3.1 [28], targeting the primary statistical test: a mixed-model ANOVA with 2 between-subjects groups (Vax vs. No-Vax), and 2 within-subjects factors—stimulus (CS+ threat-associated stimulus vs. CS− safety-associated stimulus) and time (3 SCR measurements: habituation, acquisition, extinction recall). The analysis aimed to detect a medium effect size (f = 0.25), with 95% power at an alpha level of 0.05. A moderate correlation of r = 0.5 was assumed among the six repeated measurements (2 stimuli × 3 phases), based on prior psychophysiological research and expected consistency in responses influenced by individual differences in disgust sensitivity and anxiety. This correlation was explicitly specified in GPower to ensure accurate estimation. Under the assumption of ideal sphericity (ε = 1), a minimum of 40 participants is required.

### 2.3. Inclusion Criteria

- Age range of 18–50 years.
- Right-handedness, evaluated by the Edinburgh Inventory [30].
- Normal vision.

### 2.4. Exclusion Criteria

- History of neurological or psychiatric disorders.
- Central nervous system-acting medications.
- Contraindications for Transcranial Magnetic Stimulation (TMS) [31].
- History of substance abuse or alcohol dependence.
- Participation in other neurophysiological studies within the last six months to avoid potential carryover effects.

### 2.5. Screening and Psychological Assessments

Participants will first complete an online screening questionnaire to verify eligibility. Eligible individuals will attend an in-person laboratory session, where written informed consent will be obtained prior to assessments.

- Each participant will undergo a comprehensive psychological evaluation using validated instruments designed to assess emotional, cognitive, and ideological dimensions.
- Disgust Sensitivity Scale [32]: Evaluates individual sensitivity to disgust. Individuals with high disgust sensitivity may react more strongly to perceived bodily invasions (e.g., needles, foreign substances), associating vaccines with contamination or violation of purity. This heightened aversive response can contribute to intuitive vaccine rejection rooted in pathogen avoidance and moral purity schemas.
- State-Trait Anxiety Inventory [33]: Assesses both situational (state) and dispositional (trait) anxiety levels. Elevated trait anxiety may increase health-related fears, including vaccine side effects, leading to avoidant behaviors. High state anxiety during periods of public health uncertainty (e.g., pandemics) may amplify mistrust in medical systems or increase susceptibility to fear-based misinformation.
- Revised Green et al. Paranoid Thoughts Scale [34]: Measures paranoia-related cognitive patterns. Higher levels of paranoia are associated with suspicion toward authorities, including health institutions and pharmaceutical companies. Individuals with elevated paranoid thinking may interpret public health messages as manipulative or coercive, fostering conspiratorial beliefs about vaccines.
- Interpersonal Reactivity Index [35]: Assesses empathy through Empathic Concern and Personal Distress subscales. High empathic concern might reduce hesitancy, as empathy fosters prosocial motivation, including protecting vulnerable populations through herd immunity.
- Updated Rokeach Dogmatism Scale [36]: Evaluates the degree of rigid, dogmatic thinking. High dogmatism reflects cognitive rigidity and resistance to information that contradicts pre-existing beliefs. Dogmatic individuals are less likely to accept evolving scientific recommendations and more prone to ideologically driven vaccine skepticism, especially if their belief system is anti-establishment or mistrustful of science.
- Political Orientation: Determines self-reported political inclination (liberal, centrist, or conservative) through a direct verbal question during the screening process. Moreover, we will include left-right self-placement [37]. Compared to liberal, Conservatives may show higher hesitancy in contexts where vaccines are framed as government mandates.

### 2.6. Techniques

#### 2.6.1. Experiment 1

##### Transcranial Magnetic Stimulation (TMS) and Motor Evoked Potentials (MEPs)

To determine the optimal scalp positions (OSP) for stimulation, transcranial magnetic stimulation (TMS) will be applied using a 70 mm figure-of-eight coil connected to a MagStim Super-Rapid stimulator (The Magstim Company, Wales, UK) placed over the left M1. MEPs will be recorded via a Biopac MP-36 (BIOPAC Systems, Inc., Goleta, CA, USA) electromyographic system. Ag-AgCl Self-adhesive surface electrodes (15 × 20 mm diameter) will be placed over the right deltoid (target muscle, reference over the acromion process) and the right extensor carpi radialis (ECR; control muscle, reference over the dorsal wrist). We will place the ground electrode over the right elbow. The ECR was selected as the control muscle because, like the deltoid, it is located in the upper limb, allowing for topographically matched comparisons within the same somatomotor cortical region. In the context of MEPs elicited via TMS, this is critical: upper limb muscles are represented adjacently in the lateral primary motor cortex, allowing for consistent stimulation parameters (e.g., coil orientation, hotspot location) and minimizing anatomical confounds.

The coil will be held tangentially to the scalp, with the handle oriented at approximately 45° posterolaterally to the midline, in accordance with standard procedures for optimal activation of the primary motor cortex [38,39].

The coil will be systematically shifted in 1 cm increments over the motor cortex to identify the OSP for each muscle, which will be marked on the scalp using a makeup pencil. Once the OSP is determined, the resting motor threshold (rMT) will be established, defined as the lowest stimulation intensity capable of eliciting at least five MEPs of ≥50 μV in amplitude across ten consecutive magnetic pulses. EMG signal will be continuously recorded throughout the experimental session to confirm the absence of muscular pre-activation. It will be band filtered (5 Hz–2.5 kHz, sampling rate 10 kHz), digitalized, and stored for offline analysis. To ensure comparability across participants, all MEP amplitudes will be log-transformed (log value in mV + 1) to reduce skewness. Mean MEP amplitudes for each condition will be then normalized and expressed as a % of the baseline.

**Figure 1:**
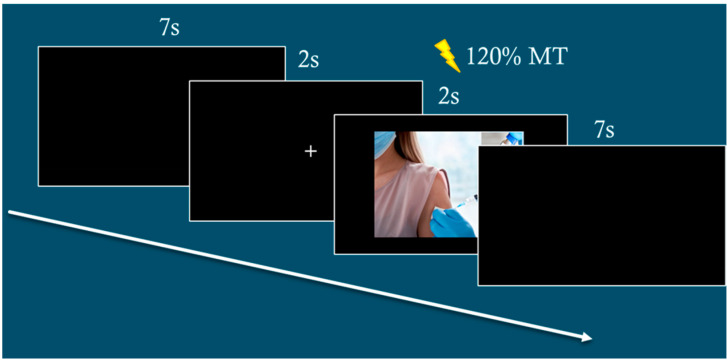
Examples of typical event trials.

MEP peak-to-peak amplitudes (in mV) will be stored for offline analysis. The inter-stimulus interval was set at 8500 ms. In this way, the TMS frequency during experimental blocks was < 0.1 Hz to avoid that TMS per se would influence M1 excitability [40]. See Figure 1 for a diagram of the typical experimental session.

The magnetic stimulation will be delivered at random times ranging between 1100 and 1400 ms from onset of the picture to avoid any priming effects that might influence MEP amplitude like in previous studies of our group [41,42,43].

##### Electromyography (EMG)

EMG signals will be recorded using a BIOPAC MP-36 system (BIOPAC Systems, Inc., Goleta, CA, USA). The signals will be amplified (×1000) and filtered with a 100–500 Hz bandpass. Electrodes will be positioned on the left side of the face, targeting the levator labii superioris (associated with disgust) and the corrugator supercilii (associated with fear), following standard placement guidelines [44]. We will remove artifacts commonly associated with facial EMG recordings. Specifically, the EMG signals will be visually inspected for non-physiological spikes, eye blinks, speech-related artifacts, or excessive movement. Trials containing artifacts (e.g., signals exceeding ±3 SD from baseline) will be excluded from further analysis. If >25% of trials from a given condition are affected, the participant will be excluded from that analysis. Finally, a baseline correction will be applied relative to a pre-stimulus window to account for tonic muscle activity.

#### 2.6.2. Experiment 2

##### Virtual Reality (VR)

To investigate the influence of immersive environments on vaccine-related attitudes, it will employ a 3D virtual reality (VR) setup. Participants will experience a simulated vaccination scenario using the Oculus Rift system (Meta Platforms Inc., Menlo Park, CA, USA), which provides high immersion through high-resolution OLED displays, a 90 Hz refresh rate, and a 110° field of view. Motion tracking capabilities will facilitate realistic interaction within the environment, while integrated 3D spatial audio will enhance sensory engagement. A dedicated graphics workstation equipped with an NVIDIA Titan X graphics card will ensure seamless rendering and optimal performance.

##### Skin Conductance Response (SCR)

Skin conductance response (SCR) will be recorded to assess autonomic nervous system activation in response to vaccination-related and unrelated stimuli. Electrodermal activity (EDA) will be measured using Ag-AgCl electrodes affixed to the middle phalanges of the non-dominant hand, with isotonic gel applied to ensure a stable signal acquisition. Data will be collected using a Biopac MP36 system with a BioNomadix module transmitter, filtered with a 1 Hz low pass setting, and processed offline using Biopac’s Acknowledge 4.3 software.

### 2.7. Interventions

#### 2.7.1. Experiment 1

**Figure 2:**
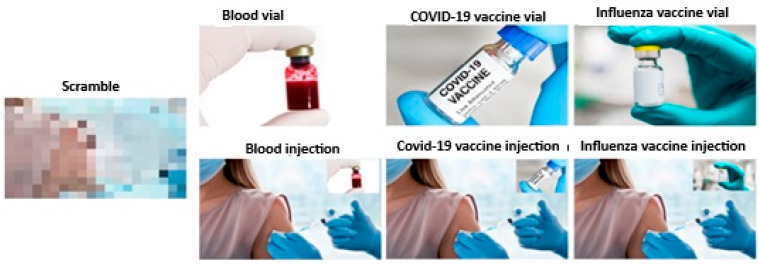
Experimental stimuli used while recording MEPs from the right DEL and ECR muscles.

Prior to the experimental session, participants will complete demographic and psychological questionnaires. Following screening, the optimal scalp positions (OSP) and resting motor threshold (rMT) for the target (deltoid) and control (extensor carpi radialis) muscles will be determined. Participants will be seated 80 cm from a computer screen in a dimly lit room. The experimental visual stimuli will consist of pictures depicting scenes of vials and ongoing injections referring to blood sampling, COVID-19 vaccine, and influenza vaccine subtending a 10.5 × 5.8 cm region plus a neutral control stimulus consisting of a scrambled picture of the same dimension and form (see Figure 2).

For each type of stimulus in both blocks, 20 MEPs will be recorded. To ensure a balanced and unbiased presentation, stimuli will be randomly presented. A total of 16 MEPs per muscle and condition will be obtained and stored for offline analysis.

During stimulus presentation, TMS pulses will be delivered at 120% of rMT at randomized intervals (1100–1400 ms post-stimulus onset) to mitigate priming effects. EMG will record muscle activity from the levator labii superioris, which is associated with disgust responses, and the corrugator supercilii, linked to fear responses. Additionally, following the TMS session, participants will be presented again with the same set of pictures and will ask to rate their emotional responses using a 7-point Likert scale, where 1 indicated no emotional reaction and 7 indicated a very strong emotional reaction. Specifically, they were asked to evaluate the extent to which each image elicited feelings of alarm, disgust, fear, and anger.

#### 2.7.2. Experiment 2

Following the completion of demographic and psychological questionnaires, participants will be immersed in a virtual reality (VR) environment for a Pavlovian fear conditioning and extinction task created by our group [45,46]. The paradigm consists of three phases—habituation, acquisition, and extinction, conducted over one day. During habituation, participants will observe two colored doors (blue and red) presented in a randomized order across eight trials. In the acquisition phase, the blue door (CS+) will be paired with an aversive stimulus—an approaching, screaming monster (80 dB)—in 80% of trials, while the red door (CS−) will not be associated with any aversive event. Ten minutes after acquisition, the extinction phase will begin. During this phase, the blue door (CS+) will no longer be followed by the aversive stimulus, allowing participants to experience its diminished threat value. Extinction will last approximately ten minutes and contain 20 trials. The recall session will occur 24 h later, during which the same blue door (CS+) will again be presented without any aversive stimulus. Throughout the experiment, participants will rate the perceived fearfulness of each stimulus using a 10-point Likert scale (1 = not scary at all; 10 = extremely scary). Typical CS− and CS+ sequences during the acquisition phase can be found in Lucifora et al. [47]. Participants scoring high on the State-Trait Anxiety Inventory will be excluded from the experiment.

### 2.8. Statistical Methods

All statistical analyses will be conducted using Jamovi (version 2.6.23), an open-source statistical software based on the R 4.5.0 framework. A significant threshold of *p* < 0.05 will be applied to all analyses. Parametric methods will be employed under the assumptions of normality and homogeneity of variance, verified by Shapiro-Wilk or Kolmogorov-Smirnov and Levene’s tests, respectively. Non-parametric tests will be used if assumptions are violated.

#### 2.8.1. Experiment 1

A 2 × 2 × 3 mixed-design ANOVA will be conducted to analyze differences in MEP amplitudes, with group (Vax vs. No-Vax) as a between-subjects factor, muscle (deltoid vs. extensor carpi radialis), condition (Blood, COVID-19, influenza), as within-subjects factors.

EMG activity from the levator labii superioris (disgust) and corrugator supercilii (fear) will be analyzed using a 2 × 2 × 3 mixed-design ANOVA, incorporating group (Vax vs. No-Vax), muscles (levator labii superioris, corrugator supercilii), condition (Blood, COVID-19, influenza).

Significant interactions (*p* < 0.05) will be followed by Tukey’s HSD post hoc tests to identify condition-specific effects.

#### 2.8.2. Experiment 2

Habituation, fear acquisition, extinction, and recall will be assessed through Skin Conductance Response (SCR) amplitudes using a 2 × 2 × 3 mixed-design ANOVA. The analysis will compare responses to stimuli (CS+ threat-associated stimulus, CS− safety-associated stimulus) as a within-subjects factor, across groups (Vax vs. No-Vax) as a between-subjects factor and phases (habituation, fear acquisition, fear extinction) as a within-subjects factor. Similarly, subjective fear/disgust ratings (Likert scale) will be analyzed using a 2 × 2 × 3 mixed-design ANOVA, with group (Vax vs. No-Vax) as a between-subjects factor, and stimulus Type (CS+ vs. CS−) and phase (habituation, fear acquisition, fear extinction) as within-subjects factors. Significant interactions (*p* < 0.05) will be followed by Tukey’s HSD post hoc tests to identify condition-specific effects.

Pearson’s or Spearman’s correlations (depending on normality) will examine associations between psychometric measures (i.e., disgust sensitivity, anxiety) and neurophysiological markers (MEPs, SCR, EMG). To address inflated Type I error rates due to multiple comparisons, all correlation analyses will be corrected using Bonferroni correction. This method adjusts the family-wise error rate by dividing the overall significance threshold (α = 0.05) by the number of comparisons performed. Hierarchical regression models will assess whether psychological variables are associated with physiological responses. Associations with VH will be explored through between-group comparisons.

## 3. Discussion

This study aims to provide novel insights into the psychophysiological correlates of vaccine hesitancy (VH). Grounded in the Somatic Marker Hypothesis [25], we hypothesize that bodily and emotional responses will play a crucial role in shaping vaccination attitudes. By investigating sensorimotor mapping, affective learning processes, and psychological predictors of VH, we seek to contribute to a more comprehensive understanding of this phenomenon.

### 3.1. Experiment 1

We predict significant differences in motor evoked potentials (MEPs) between vaccine-hesitant and vaccine-accepting individuals when exposed to COVID-19 vaccination imagery. Specifically, hesitant individuals may exhibit reduced MEP amplitudes in the deltoid muscle—a key muscle involved in vaccine administration—suggesting altered sensorimotor resonance in response to vaccination stimuli. This would align with previous research on embodiment, which posits that motor responses are modulated by observed actions [48,49]. The sensorimotor resonance may serve as a proxy for embodied affective states such as disgust or fear—emotions that are central to the Somatic Marker Hypothesis, which emphasizes the role of visceral and emotional signals in guiding behavior and cognition [25]. Therefore, a decrease in MEP amplitude may indicate a disruption of somatic marker signals, given that corticospinal excitability is sensitive to modulation by negative emotional states. This interpretation is supported by prior work demonstrating the influence of emotions—particularly aversive or defensive states on motor system activity [39,40,41,50,51,52,53]. We suggest that motor resonance changes, in this context, may index the embodied affective experience central to the SMH, thereby linking motor excitability to emotional and interoceptive processing.

Additionally, we anticipate increased facial electromyography (EMG) activity in the levator labii superioris and corrugator supercilii muscles in vaccine-hesitant individuals, reflecting higher levels of disgust and fear responses. If confirmed, these results would support the hypothesis that VH is influenced by affective biases linked to disgust sensitivity and anxiety [9,21].

### 3.2. Experiment 2

Through the Pavlovian fear conditioning and extinction paradigm, we expect vaccine-hesitant individuals to exhibit higher skin conductance responses (SCR) to the conditioned stimulus (CS+), indicating exaggerated fear responses during the acquisition phase. Furthermore, we anticipate a lower extinction learning, leading to increased fear retention during the recall session. Such findings would suggest that vaccine-hesitant individuals exhibit altered patterns of threat updating, which could be associated with the persistence of avoidance-related behaviors. These results would align VH with other avoidance-based behaviors, emphasizing the need for interventions targeting fear extinction [22,54]. Consistent with previous research, we anticipate that specific psychological traits will be significantly associated with higher levels of VH. Higher scores in disgust sensitivity, paranoia, and dogmatic thinking are expected to be associated with increased VH. These findings would further emphasize the complex interplay between cognitive rigidity, emotional processing, and vaccine decision-making [19,20,55].

In conclusion, this protocol will have significant public health implications by advancing our understanding of the psychophysiological mechanisms underlying VH. It will offer valuable insights for future pandemics and help develop tailored public health messages that integrate sociocultural, clinical, and neurobiological factors.

## Abbreviations

The following abbreviations are used in this manuscript:

COVID-19	Coronavirus Disease 2019
VH	Vaccine Hesitancy
MEPs	Motor Evoked Potentials
EMG	Electromyography
SCR	Skin Conductance Response
WHO	World Health Organization
Vax	Vaccinated individuals
No-Vax	Non-Vaccinated individuals
TMS	Transcranial Magnetic Stimulation
IQ	Intelligence Quotient
OSP	Optimal Scalp Positions
ECR	Extensor Carpi Radialis
rMT	Resting Motor Threshold
μV	Microvolt
mV	Millivolt
Hz	Hertz
3D	Three Dimensional
VR	Virtual Reality
EDA	Electrodermal Activity
dB	Decibel
ANOVA	Analysis of Variance
CS+	Conditioned Stimulus Positive
CS-	Conditioned Stimulus Negative
Tukey’s HSD	Tukey’s Honestly Significant Difference
